# Thermal Charging Optimization of a Wavy-Shaped Nano-Enhanced Thermal Storage Unit

**DOI:** 10.3390/molecules26051496

**Published:** 2021-03-09

**Authors:** Mohammad Ghalambaz, S.A.M. Mehryan, Ahmad Hajjar, Mohammad Yacoub Al Shdaifat, Obai Younis, Pouyan Talebizadehsardari, Wahiba Yaïci

**Affiliations:** 1 Metamaterials for Mechanical, Biomechanical and Multiphysical Applications Research Group, Ton Duc Thang University, Ho Chi Minh City 758307, Vietnam; mohammad.ghalambaz@tdtu.edu.vn; 2 Faculty of Applied Sciences, Ton Duc Thang University, Ho Chi Minh City 758307, Vietnam; 3 Young Researchers and Elite Club, Yasooj Branch, Islamic Azad University, Yasooj 7591493686, Iran; alal171366244@gmail.com; 4 ECAM Lyon, LabECAM, Université de Lyon, 69005 Lyon, France; ahmad.hajjar@ecam.fr; 5 Department of Mechanical and Manufacturing Engineering, Universiti Kebangsaan Malaysia, Bangi 43600, Malaysia; shd.mo92@gmail.com; 6 Department of Mechanical Engineering, College of Engineering at Wadi Addwaser, Prince Sattam Bin Abdulaziz University, Wadi Addwaser 11991, Saudi Arabia; oubeytaha@hotmail.com; 7 Department of Mechanical Engineering, Faculty of Engineering, University of Khartoum, Khartoum 11111, Sudan; 8 Canmet ENERGY Research Centre, Natural Resources Canada, 1 Haanel Drive, Ottawa, ON K1A 1M1, Canada

**Keywords:** wavy-tube, nano-enhanced phase change material (NePCM), latent heat thermal energy storage (LHTES) unit, charging time

## Abstract

A wavy shape was used to enhance the thermal heat transfer in a shell-tube latent heat thermal energy storage (LHTES) unit. The thermal storage unit was filled with CuO–coconut oil nano-enhanced phase change material (NePCM). The enthalpy-porosity approach was employed to model the phase change heat transfer in the presence of natural convection effects in the molten NePCM. The finite element method was applied to integrate the governing equations for fluid motion and phase change heat transfer. The impact of wave amplitude and wave number of the heated tube, as well as the volume concertation of nanoparticles on the full-charging time of the LHTES unit, was addressed. The Taguchi optimization method was used to find an optimum design of the LHTES unit. The results showed that an increase in the volume fraction of nanoparticles reduces the charging time. Moreover, the waviness of the tube resists the natural convection flow circulation in the phase change domain and could increase the charging time.

## 1. Introduction

The major threat in energy society is the expanding gap between the supply of energy and global demand, which makes it challenging for engineers to find unique solutions to fulfill the needs of this society. The problems on energy conservation and storage were under the scientific community’s concern since figuring out the idea of utilizing the industrial hot waste streams and the abundant-solar thermal energy [1]. The latent heat is an alternative to store thermal energy in the form of latent heat of phase change through a melting or freezing process [2]. The classifications of phase change materials (PCMs) include eutectics, organic, and inorganic materials. Most studies focus on organic materials due to their large heat storage capacity, different phase-change temperatures, and low cost [3,4]. A PCM for thermal energy storage was prepared by Osterman et al. [5] for heating and cooling purposes to save energy. Their study showed annual save reaches 142 kWh in the energy consumption of an office.

Substitutes for PCM have been developed recently by the composite metal foam/PCM to enhance the heat transfer inside PCM [6,7]. A study by Mesalhy et al. [8] showed that the porous foam matrix within PCM has a significant impact on the rate of heat transfer and charging time. Sardari et al. [2] made a numerical simulation to evaluate the discharging mechanism in a phase change material to air heat exchanger, which aimed to have space heating using PCM and composite of copper foam. Significant advantages were shown in the result. By using the composite PCM, a 73% higher heat retrieval rate was achieved, and the solidification time was reduced by 43%. The low thermal conductivity is considered a well-known property of organic PCMs. This property leads to a limitation in absorbing and releasing heat. Therefore, nanoparticles provide good physical and chemical properties to the PCMs compared to their bulk form [9,10]. Achieving higher thermal conductivity is related to the reduction in nanoparticle size [11]. A new sort of nano-enhanced phase change material (NePCM) was developed by Wu et al. [12] by suspending a small amount of C/Cu, Cu, and Al nanoparticles paraffin to improve the heat transfer rate of paraffin. The study found that Cu nanoparticles have the best heat transfer performance.

Chen et al. [13] added a small amount of CuO nanoparticles to paraffin to improve thermal storage and solar thermal conversion. The nanoparticles impressively improved the light abortion and solar energy conversion. The nanoparticles could improve the steady temperature of the unit up to 2.3 times compared to the pure paraffin.

Developing additives is an interesting task that has been advanced by the synthesis of novel nanoadditives. Successful utilization of NePCM in heat transfer applications depends on some important terms, which can be summarized as follows [14]: (1) on the level of required flexibility, thermal stability, strength, and corrosion resistance. (2) Protection shield for the PCM to avoid any harmful interaction with the environment. (3) Make a sufficient heat transfer surface. (4) Provide easy handling and structural stability.

One of the effective and economical ways for heat transfer enhancement is improving the heat transfer area by using fins and extended surfaces. However, fins could obstruct the free convection circulation in molten regions [15]. Hence, a smooth development of convection by employing fins can only be achieved by optimized fin size [16]. A combination of using nanoparticles and an optimum structure of fin could avoid the disadvantage of employing nanoparticles and fins in a conical shaped latent heat thermal energy storage (LHTES) system. Singh et al. [15] used a combination of active (fins and nanoparticles) heat transfer enhancement techniques to make an innovative LHETS, where the volume of the fins was fixed (0.15) for all cases. The study found a 57% reduction in charging time of LHTES with a conical shape in the presence of 5% graphene nano-plates. On the other side, no substantial improvement was observed in thermal performance of finned LHTES with 1% GNP (graphene nanoplatelets) because of the restriction of natural convection vortices and raised viscosity. Different studies found that the geometry of PCM-based heat storage units influences heat transfer performance such as the number and the shape of heat transfer fluid (HTF) inner tubes [17]. Mehta et al. [18] utilized different tubes in an LHTES, i.e., multiple-tubes, the eccentricity of heat transfer fluid (HTF) tube, and rotation of the HTF tube. The study concluded that the maximum reduction in the melting time was achieved by rotating latent heat storage (LSH) systems with a 47.75% reduction. Wavy walls are considered a promising technique on heat transfer enhancement instead of straight walls by mixing up the flow in both sides of the wavy wall and increasing the surface area of heat transfer [19,20,21,22]. Shahsavar et al. [23] investigated the solidification and melting performance in a wavy LHTES with composite PCM and copper foam. They showed that a wavy enclosure could have promising advantages in the system, where strong influences of metal foams suppressed the convection heat transfer.

The literature studies show the impact of wavy walls on the convective and conductive heat transfer flows. Thus, the present study aims to address the impact of using wavy tubes in shell and tube heat thermal energy storage for the first time. The current research aims to investigate the advantage of using wavy tubes in clear flows (with no porous metal foam) where the convective heat transfer effects are important. The impact of using nanoadditives on the charging time of the energy storage system will also be investigated.

## 2. Mathematical Model

**Figure 1:**
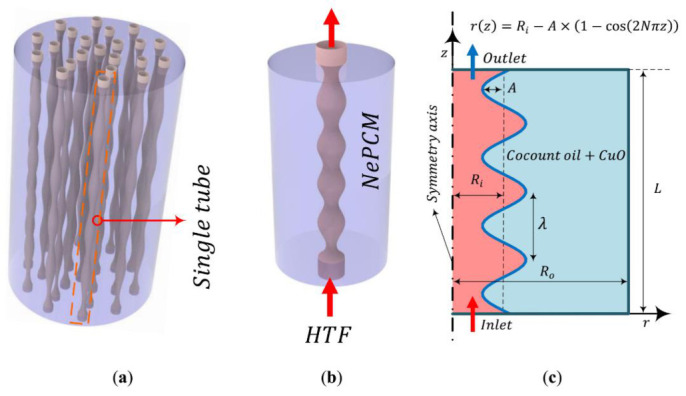
The model of the energy storage system: (**a**) latent heat thermal energy storage (LHTES) unit, (**b**) single tube with the nano-enhanced phase change material (NePCM) domain; (**c**) computational domain.

A multi-tube shell-and-tube LHTES unit, in which the NePCM is arranged in the shell side with an initial super cold temperature of *T_initial_*, and the hot fluid flows through the wavy tubes, as shown in Figure 1a. The tubes are placed next to each other. Here, one of the tubes (see Figure 1b) is selected for the investigation of melting heat transfer. The HTF with a high temperature is injected into the wavy tube with a temperature of *T_in_*, the heat is transferred between the wavy wall and the NePCM, with thermal energy stored in the NePCM. A 2D axis symmetric view of the model is shown in Figure 1c. 

The PCM and the nanoadditives on the NePCM are the coconut oil and CuO nanoparticles, with the thermophysical characteristics listed in Table 1. The length of the system, i.e., *L*, is 100 mm, the outer radius of the computational domain, i.e., *R_o_*, is 50 mm, and the radius of the simple tube, i.e., *R_i_*, is 9 mm. Assuming the tube is sufficiently thin and high-thermally conductive, the temperature gradient and energy stored can be ignored in the tube. The amplitude and period of the wavy wall are A and N, respectively. The properties of the HTF flowing in the tube, i.e., water, are tabulated in Table 1.

### 2.1. Convective Phase Change Heat Transfer in NePCM

The NePCM melts over time, and the natural convection of the molten NePCM occurs in the liquid phase. The transient phase change process with natural convection is described using the volume-averaged approach:

(I) Mass conservation

(1) $$\frac{1}{r}\frac{\partial \left(ru_{r}\right)}{\partial r}+\frac{\partial u_{z}}{\partial z}=0$$

where *r* and *z* are radial and axial directions while *u_r_* and *u_z_* are radial and axial velocity components.

(II) Momentum equations

(2) $$\begin{array}{l} \rho_{NP,l}\left(\frac{\partial u_{r}}{\partial t}+u_{r}\frac{\partial u_{r}}{\partial r}+u_{z}\frac{\partial u_{r}}{\partial z}\right)=-\frac{\partial p}{\partial r}+\frac{\mu_{NP,l}}{r}\frac{\partial }{\partial r}\left(r\frac{\partial u_{r}}{\partial r}\right)-\frac{\mu_{NP,l}u_{r}}{r^{2}}\\ +\mu_{NP,l}\frac{\partial^{2}u_{r}}{\partial z^{2}}+A_{mush}\frac{1-2\gamma \left(T\right)+\gamma^{2}\left(T\right)}{\lambda +\gamma^{3}\left(T\right)}u_{r} \end{array}\;\begin{array}{l} \rho_{NP,l}\left(\frac{\partial u_{z}}{\partial t}+u_{r}\frac{\partial u_{z}}{\partial r}+u_{z}\frac{\partial u_{z}}{\partial z}\right)=-\frac{\partial p}{\partial z}+\frac{\mu_{NP,l}}{r}\frac{\partial }{\partial r}\left(r\frac{\partial u_{z}}{\partial z}\right)+\mu_{NP,l}\frac{\partial^{2}u_{z}}{\partial z^{2}}\\ +g\rho_{NP,l}\beta_{NP,l}\left(T-T_{me}\right)+A_{mush}\frac{1-2\gamma \left(T\right)+\gamma^{2}\left(T\right)}{\lambda +\gamma^{3}\left(T\right)}u_{z} \end{array}$$

where *g*, *μ*, *ρ*, and *β* are the gravity acceleration, dynamic viscosity, density, and thermal expansion coefficient, respectively. The filed variables of *p* and *T* are the pressure and temperature, respectively while *t* is time. The subscripts l, NP, and me refer to liquid, NePCM, and fusion temperature, respectively. NePCM. Terms containing *A_mush_* are applied to damp the velocity vector in the solid phase. The numerical constant *A_mush_* and *λ* are relatively large (5 × 10^5^ kg/(m^3^s)) and small (10^−3^), respectively. The function $\gamma \left(T\right)$ of the above equations is described as the following:

(3) $$\gamma \left(T\right)=\left\{\begin{array}{cccc} 0 & & & T<T_{me}-\Delta T_{me}/2\\ \frac{T-T_{me}}{\Delta T_{me}}+\frac{1}{2} & & & T_{me}-\Delta T_{me}/2<T<T_{me}+\Delta T_{me}/2\\ 1 & & & T>T_{me}+\Delta T_{me}/2 \end{array}\right.,$$

where *T* is temperature and Δ*T_me_* is the melting range.

(III) Energy conservation

(4) $$\begin{array}{l} {\left(\rho C_{p}\right)}_{NP}\frac{\partial T}{\partial t}+(\rho C_{p}{)}_{NP,l}\left(u_{r}\frac{\partial T}{\partial r}+u_{z}\frac{\partial T}{\partial z}\right)=\frac{1}{r}\frac{\partial }{\partial r}\left(k_{NP}r\frac{\partial T}{\partial r}\right)\\ +\frac{\partial }{\partial z}\left(k_{NP}\frac{\partial T}{\partial z}\right)-\left(1-\omega_{na}\right)\rho_{PCM,l}h_{f,PCM}\frac{\partial \omega \left(T\right)}{\partial t} \end{array},$$

in which *k*, *h_f_*, *C*_p_, and *ω_na_* are the thermal conductivity, specific heat capacity, latent heat, and volume concentration of nanoparticles, respectively. The subscripts *PCM* and *na* show the pure PCM and nanoparticles. In the above equations, the property of NePCM is the weighted average of its liquid and solid states, depending on the melt fraction *γ*(*T*), which are evaluated as:

(5) $$\begin{array}{l} {\left(\rho C_{p}\right)}_{NP}=\gamma \left(T\right)\left[{\left(\rho C_{p}\right)}_{NP,l}-{\left(\rho C_{p}\right)}_{NP,s}\right]+{\left(\rho C_{p}\right)}_{NP,s}\\ k_{NP}=\gamma \left(T\right)\left(k_{NP,l}-k_{NP,s}\right)+k_{NP,s} \end{array},$$

where subscript *s* indicates the solid state.

### 2.2. The Properties Model

The NePCM is a mixture of PCM and nanoparticles. Thus, following the conservation of mass, the density can be evaluated as:

(6) $$\begin{array}{l} \rho_{NP}=\rho_{PCM}+\omega_{na}\left(\rho_{na}-\rho_{PCM}\right)\\ \rho_{PCM}\left(T\right)=\gamma \left(T\right)\left(\rho_{PCM,l}-\rho_{PCM,s}\right)+\rho_{PCM,s} \end{array},$$

in which ω*_na_* denotes the volumetric concertation of CuO nanoparticles. The Brinkman model is applied to evaluate the liquid NePCM’s dynamic viscosity as:

(7) $$\mu_{NP,l}=\mu_{PCM,l}{\left(1-\omega_{na}\right)}^{-2.5},$$

The volumetric expansion of NePCM is important for the evaluation of buoyancy forces. This coefficient for the NePCM mixture is evaluated as:

(8) $$\rho_{NP,l}\beta_{NP,l}=\rho_{PCM,l}\beta_{PCM,l}+\omega_{na}\left(\rho_{na}\beta_{na}-\rho_{PCM,l}\beta_{PCM,l}\right),$$

The Maxwell relation is employed to estimate the NePCM’s thermal conductivity as [26]:

(9) $$\frac{k_{NP,i}}{k_{PCM,i}}=\frac{\left(k_{na}+2k_{PCM,i}\right)-2\omega_{na}\left(k_{PCM,i}-k_{na}\right)}{\left(k_{na}+2k_{PCM,i}\right)+\omega_{na}\left(k_{PCM,i}-k_{na}\right)},$$

Equation (9) was discussed in [26] and showed good agreement with experimental measurement for 15–25 nm spherical nanoparticles. Finally, the NePCM’s heat capacity was computed as:

(10) $$\begin{array}{l} {\left(\rho C_{p}\right)}_{NP}=\rho_{PCM}C_{p,}{}_{PCM}+\omega_{na}\left(\rho_{na}C_{p}{}_{,na}-\rho_{PCM}C_{p,}{}_{PCM}\right)\\ \rho_{PCM}C_{p,}{}_{PCM}\left(T\right)=\gamma \left(T\right)\left[\rho_{PCM,l}C_{p}{}_{,PCM,l}-\rho_{PCM,s}C_{p}{}_{,PCM,s}\right]+\rho_{PCM,s}C_{p}{}_{,PCM,s} \end{array},$$

### 2.3. Convective Heat Transfer in HTF

Assuming the *HTF* injected in the tubes is laminar, transient, and incompressible, the convective heat transfer can be described by the below equations:

(I) Conservation mass

(11) $$\frac{1}{r}\frac{\partial \left(ru_{r}\right)}{\partial r}+\frac{\partial u_{z}}{\partial z}=0,$$

(II) Momentum equations

(12) $$\begin{array}{l} \rho_{HTF}\left(\frac{\partial u_{r}}{\partial t}+u_{r}\frac{\partial u_{r}}{\partial r}+u_{z}\frac{\partial u_{r}}{\partial z}\right)=-\frac{\partial p}{\partial r}+\frac{\mu_{HTF}}{r}\frac{\partial }{\partial r}\left(r\frac{\partial u_{r}}{\partial r}\right)-\frac{\mu_{HTF}u_{r}}{r^{2}}+\mu_{HTF}\frac{\partial^{2}u_{r}}{\partial z^{2}}\\ \rho_{HTF}\left(\frac{\partial u_{z}}{\partial t}+u_{r}\frac{\partial u_{z}}{\partial r}+u_{z}\frac{\partial u_{z}}{\partial z}\right)=-\frac{\partial p}{\partial z}+\frac{\mu_{HTF}}{r}\frac{\partial }{\partial r}\left(r\frac{\partial u_{z}}{\partial r}\right)+\mu_{HTF}\frac{\partial^{2}u_{z}}{\partial z^{2}} \end{array}$$

(III) Energy conservation

(13) $${\left(\rho C_{p}\right)}_{HTF}\left(\frac{\partial T}{\partial t}+u_{r}\frac{\partial T}{\partial r}+u_{z}\frac{\partial T}{\partial z}\right)=\frac{1}{r}\frac{\partial }{\partial r}\left(k_{HTF}r\frac{\partial T}{\partial r}\right)+\frac{\partial }{\partial z}\left(k_{HTF}\frac{\partial T}{\partial z}\right),$$

where the subscript *HTF* refers to heat transfer fluid.

### 2.4. Controlling Boundary and Initial Conditions

The temperature and heat continuity are adopted for the thermal boundary condition at the surface of the wavy tube as [27,28]:

(14) $${\left.T\right|}_{\;HTF}={\left.T\right|}_{NP},\;{\left.k_{HTF}\frac{\partial T}{\partial n}\right|}_{HTF}{}_{\;}={\left.k_{NP}\frac{\partial T}{\partial n}\right|}_{NP},$$

The HTF is injected with the temperature of *T_in_* and *v_in_*.

(15) $${\left.T\right|}_{\;HTF}=T_{in},\;{\left.u_{r}\right|}_{\;HTF}=0,{\left.u_{z}\right|}_{\;HTF}=u_{z,in},$$

where *T_in_* is the inlet temperature for HTF. At the outlet of the HTF tube, the developed convective flow is considered to be established.

(16) $${\left.u_{r}\right|}_{\;HTF}=0,\;{\left.\frac{\partial T}{\partial z}\right|}_{HTF}{}_{\;}={\left.\frac{\partial u_{z}}{\partial z}\right|}_{HTF}=0,$$

Due to the symmetrical distribution of the tube bundle, the boundary conditions of the computational domain are:

(17) $${\left.u_{r}\right|}_{\;NP}={\left.u_{z}\right|}_{\;NP}=0,\;{\left.\frac{\partial T}{\partial r}\right|}_{NP}{}_{\;}=0,$$

At the upper and lower surfaces of the tube bundle, the boundary conditions can be defined as the following:

(18) $${\left.u_{r}\right|}_{\;NP}={\left.u_{z}\right|}_{\;NP}=0,\;{\left.\frac{\partial T}{\partial z}\right|}_{NP}{}_{\;}=0,$$

The initial condition for the NePCM domain can be expressed as the following:

(19) $${\left.u_{r}\right|}_{\;NP}={\left.u_{z}\right|}_{\;NP}=0,\;{\left.T\right|}_{NP}{}_{\;}=T_{initial},$$

where in the above equation *T_in_*= 323 K, and *T_initial_* = 293 K.

### 2.5. Heat Transfer Characteristics

The cumulative heat transferred ($ES\left(t\right)$) from the HTF to the NePCM is exactly the stored energy (*ES*) in the unit. $ES\left(t\right)$ can be calculated by integrating the instantaneous heat transfer rate from the wavy wall:

(20) $$ES\left(t\right)=\int_{A}\left[\int_{T_{initial}}^{T}{\left(\rho C_{p}\right)}_{NP}dT\right]\;dA+\int_{A}\left(1-\omega_{na}\right)\rho_{PCM,l}h_{f,PCM}dA,$$

In Equation (21), the first term shows the sensible heat stored in the NePCM, and the second term takes into account the latent heat stored in the NePCM. Moreover, the rate of the absorbed heat by NePCM, Δ*q*, can be computed as:

(21) $$\Delta q=E_{2}-E_{1}\;∵\;E_{1}=\pi R_{i}^{2}\rho_{HTF}u_{z,in}C_{p,HTF}T_{in}\;\mathrm{and}\;\;E_{2}=\pi \rho_{HTF}C_{p,HFT}\int_{0}^{R_{i}}u{\;}_{z}T\;rdr$$

Moreover, the average temperature of the interface surface can be evaluated as:

(22) $$\;{\bar{T}}_{s}=\int_{0}^{s}T_{s}\;ds/\int_{0}^{s}ds,$$

The average convective heat transfer coefficient ($h_{HTF}$) at the water side can be computed by using Newton’s cooling law as:$\;\;\Delta q=2\pi R_{i}\times L\times h_{HTF}\times \left(T_{in}-{\bar{T}}_{s}\right)$ where the inlet temperature and the enclosure high were considered as the reference temperature and the characteristic length, respectively. Thus, $h_{HTF}$ is computed as:

(23) $$h_{HTF}=\;\;\frac{\Delta q}{2\pi R_{i}L\left(T_{in}-{\bar{T}}_{s}\right)}$$

Using the same approach, the heat transfer coefficient ($h_{NP}$) at the NePCM side can also be computed as following. By neglecting small unsteady temperature fluctuations at the water side, the transported energy from the water to the NePCM is equal to stored energy in the NePCM. Thus, $h_{PCM}$ can be introduced as:

(24) $$h_{NP}=\;\;\frac{\Delta q}{2\pi R_{i}L\left({\bar{T}}_{s}-T_{me}\right)},$$

Here, *L* was taken as the characteristic length of the wavy tube instead of the arc length *s*. This is because the variation of amplitude and the wave number of the wavy pipe could change the arc length, and hence, the using arc length *s* would lead to the convection heat transfer directly dependent on the multiplication of heat transfer coefficient and the actual surface of heat transfer. However, since *L* is constant, the computed values of *h* can be directly compared and judged. Moreover, the thermal resistance can be computed as:

(25) $$\begin{array}{l} R_{HTF}=\frac{1}{2\pi R_{i}Lh_{HTF}}\;\mathrm{at}\;\mathrm{the}\;\mathrm{HTF}\;\mathrm{side},\\ R_{NP}=\frac{1}{2\pi R_{i}Lh_{NP}}\mathrm{at}\;\mathrm{the}\;\mathrm{NePCM}\;\mathrm{side}, \end{array}$$

Furthermore, the pressure loss of the HTF passing the channel can be expressed as the following:

(26) $$\Delta p_{HTF}=2\pi \left(\int_{0}^{R_{i}}pdr\left|{}_{inlet}\right.-\int_{0}^{R_{o}}pdr\left|{}_{outlet}\right.\right),$$

the total Melting Volume Fraction (MVF) is evaluated as:

(27) $$MVF\left(t\right)=\frac{\int_{A}^{}\gamma \left(T\right)dA}{\int_{A}^{}dA},$$

In the practical application of the LHTES unit, the performance of the unit can be evaluated by charging power. This parameter shows the capacity of the PCM to store energy and depends on the amount of the energy stored at 100% melting volume fraction:

(28) $$CP=\frac{\mathrm{ES}\;\mathrm{when}\;\mathrm{the}\;\mathrm{melting}\;\mathrm{process}\;\mathrm{complete}}{\mathrm{complete}\;\mathrm{melting}\;\mathrm{time}},$$

## 3. Numerical Approach and Mesh Study and Verifications

Since the configurations of a single tube along with the surrounding NePCM is axisymmetric, a two-dimensional (2D) configuration can be considered to save computational time, as shown in Figure 1c. As previously discussed, in detail, to model the melting front, the enthalpy-porosity method is employed. The time-dependent finite element method involving the Galerkin approach is employed to solve the temperature, pressure, and velocity fields. Convergence is obtained for each simulation when scaled residuals are less than 10^−3^ for mass, momentum, and energy conservation equations. The influence of mesh size on the accuracy of the results is examined through a mesh sensitivity analysis for MVF. A structured mesh due to efficiency and simplicity is used to discretize the domains. Naturally, an unstructured grid requires more memory than a structured grid with an equal number of elements. As tabulated in Table 2, the liquid fractions of NePCM for five grids with different elements count are compared. Through the relative errors, it is found that a mesh with a size of 30 × 150 elements can be adequate for the purpose of optimizing the mesh elements.

**Figure 2:**
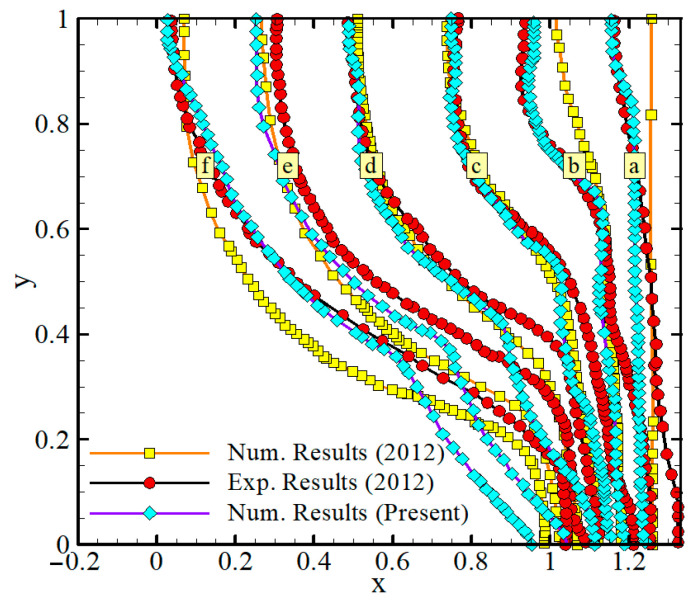
The experimental findings of Kumar et al. [29] and the numerical computations of the present work following times (s); (**a**): 139, (**b**): 277, (**c**): 416, (**d**): 554, (**e**): 693, and (**f**): 831.

**Figure 3:**
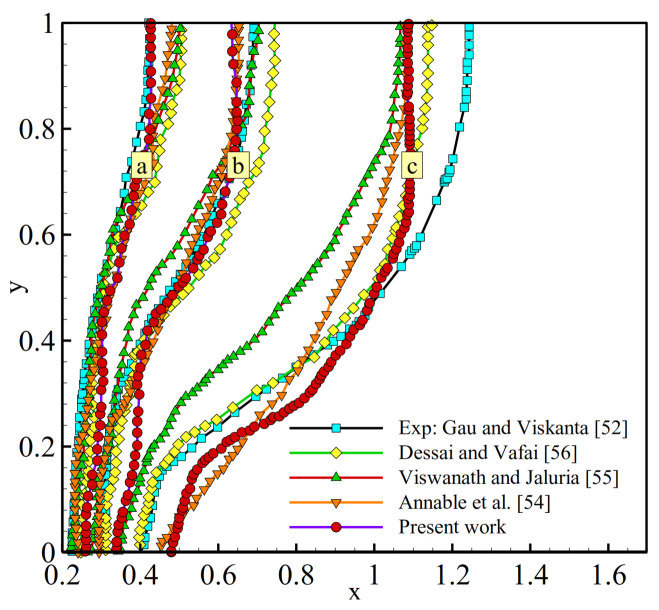
Melting lines for several studies in literature and present work for (**a**): *t* = 5 min, (**b**): 10 min, and (**c**): 19 min.

The results of the model discussed above are compared with the numerical and experimental results reported in the literature [29,30,31,32,33]. Kumar et al. [29] experimentally and numerically studied the fusion process of lead contained in a cuboid exposed a constant heat flux at the right sidewall. The dimensions of the cuboid were 50 mm in height, 50 mm in width, and 60 mm in depth. Figure 2 illustrates the progress of the melting front in the experimental and numerical study conducted by Kumar et al. [29] and the present numerical model solved by FEM. Gau and Viskanta [30] experimentally analyzed the buoyancy-driven flow in the melted pure PCM and its impact on the melting front motion. In this experiment, the melting process was followed in a rectangular test cell with a heat source located on the left sidewall and a heatsink on the opposite sidewall. The dimensions of the test cell used were 63.5 mm in width, 88.9 mm in height, and 38.1 mm in depth. Figure 3 depicts a set of progressive solid-liquid interfaces of the numerical studies compared with the experimental interfaces observed by Gau and Viskanta. There are good agreements between the results of the melting front location in the current study and the results of other works [29,30,31,32,33]. Hence, the utilized code is reliable for parametric investigation. Although it would not provide the exact values of the physical quantities, it can provide the effect of each parameter on the system.

## 4. Results and Discussion

### 4.1. Taguchi Analysis

Taguchi’s technique is a powerful statistical method utilized to optimize designs, in which the results are influenced by different parameters or control factors. Each of these control factors is assigned a number of numerical values at different levels. Taguchi’s method is then applied in order to find a minimal number of test cases required to determine the optimal values of the control factors. This is more efficient than performing all the possible tests, which presents a high cost in time and resources and can be practically very difficult to implement.

In the present study, the key parameters of the problem are the volume fraction of the nanoadditives ($0\leq \omega_{na}\leq 0.05$), the amplitude of the wavy wall (0≤A≤3mm), the period of the wavy wall (1≤N≤5). These parameters represent the control factors, and each is assigned four levels. Table 3 summarizes the control factors of the problem and their correspondent levels. If all the possible combinations of the control factors and their levels were to be tested, 4^3^ tests would be required.

The L16 orthogonal array is then used, and Taguchi’s algorithm is applied in order to reduce to16 the number of required tests. The range of the control factors and their levels corresponding to these 16 tests are listed in Table 4. In this study, the computation continued until the unit reaches full melting, i.e., MVF = 1.

As previously mentioned, the aim of Taguchi’s method is to determine the values of the control factors that would lead to an optimal result. In this regard, based on the desired result, one of the following three approaches can be used: the larger—the better, the nominal—the better, and the smaller—the better. In the present study, the time required for complete PCM melting is considered as the optimization result. As the objective is to reduce the full melting time, then the smaller—the better approach is adopted. The signal to noise ratio, SNR, is analyzed for each test. SNR represents the advantage of wanted or desired results to the undesired results. Moreover, a linear regression model is developed in order to relate the full melting time to the control factors:

Full Melt time (s) = 26,487 − 63,754 *ω_na_* + 1742.3 *A* − 17.9 *N*, (29)

After determining the combination of parameters that lead to the lowest full melting time among the tests, the optimal values of the control factors can be defined. These values are presented in Table 5: ω*_na_* = 0.06 (level 4), *A* = 0 (level 1), and *N* = 4 (level 4) with a full melting time of 22,709 s. Since *A* = 0, the value of *N* is meaningless, and indeed, the Taguchi method shows that a plane tube with no wave could lead to better performance compared to a wavy tube.

**Figure 4:**
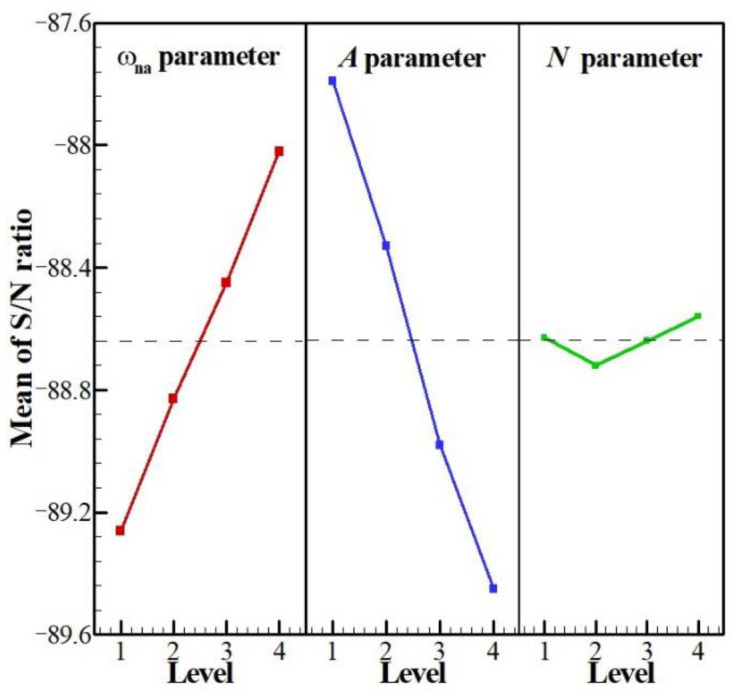
Mean values of the S/N ratios for all the levels of the controlling parameters: optimum levels: *A* = 4, *B* = 1, and *C* = 4.

The next step is to define which control factors have the most influence and the results. To do so, the SNR of the tests are analyzed. The mean values of the SNR for each test are plotted in Figure 4, while Table 6 list the values of *Δ* indicating the difference between the maximum and minimum of the mean SNR in each case. It is evident from Figure 4 that the optimal values previously obtained lead to the highest mean value of SNR among the tested parameters. *Δ* is an indicator of the degree of influence of each control factor, i.e., the higher the value of *Δ*, the greater the influence of the corresponding parameter on the target-parameter. Thus, based on Table 6, the various control factors can be classified in the order of their influence: *A* > *ω_na_* *> N*.

To further confirm the design optimization with the previously determined values, nine more tests are conducted with combinations of the design parameters around the optimal values. In each group test, two of the three control factors are kept constant at their optimal value, while the third is varied among three different values. The results show that the full melting time in all the test cases is greater than 22,709 s, which corresponds to the optimal values. This confirms that the design is optimized for the values of the control factors previously found: ω*_na_* = 0.06, *A* = 0, and *N* = 4.

### 4.2. Parametric Investigation

As the optimal values are determined, the next step is to analyze the flow and thermal patterns in the domain in order to provide physical insights explaining the aforementioned outcomes. Table 7 shows nine selected designs around the optimum case. Cases 1–3, 4–6, and 7–9 aim to investigate the impact of concentration of nanoparticles, wave amplitude, and wave number on the melting process, respectively.

**Figure 5:**
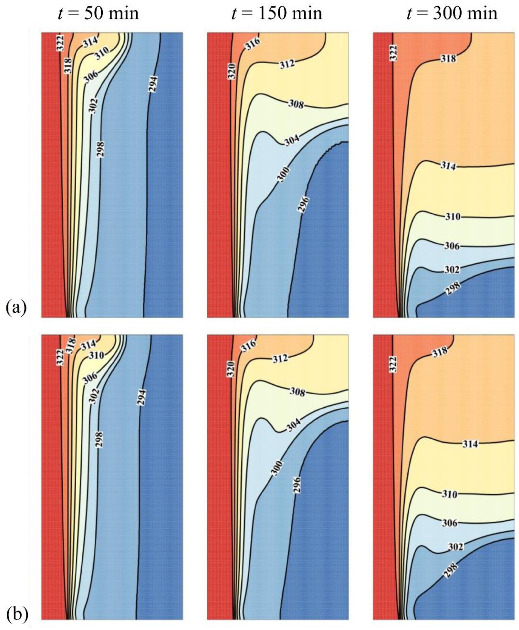
Effect of volume fraction of nanoparticles (ω*_na_*) on the contours of isotherm, (**a**) optimum case with ω_na_ = 0.06; (**b**) Case 1 with ω_na_ = 0 for *A* = 0 mm.

**Figure 6:**
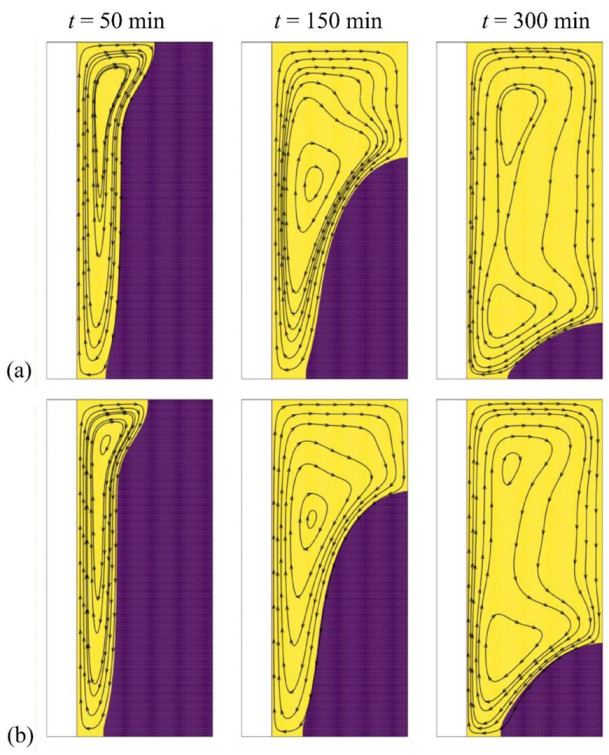
Effect of volume fraction of nanoparticles (ω*_na_*) on the streamlines, (**a**) optimum case with ω*_na_* = 0.06; (**b**) Case 1 with ω*_na_* = 0, for *A* = 0 mm.

Figure 5 and Figure 6 depict the isothermal contours and the streamlines in the cavity for two values of the nanoparticle volume fraction, ω*_na_* = 0.06 corresponding to the optimal case and ω*_na_* = 0 corresponding to pure PCM without nanoparticles. The presence of streamlines indicates that the PCM is in the liquid phase; i.e., it has melted in that region. Initially, the PCM starts to melt near the heated tube at the left. In that region, the conductive effects are important. As time goes, the hot melted PCM circulates upwards due to the density difference, and free convection takes place.

**Figure 7:**
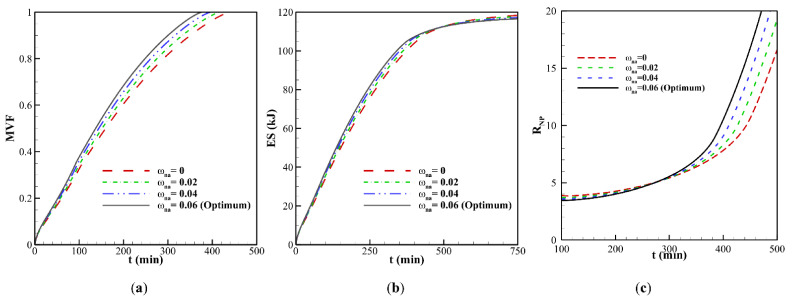
Effect of volume fraction (ω*_na_*) for *A* = 0 mm. on the: (**a**) melting volume fraction; (**b**) total energy storage; (**c**) thermal resistance at NePCM side.

A clockwise vortex is created in the domain. The hot PCM impinging in the top region transfers its heat to the melting front so that the convective effects, and consequently PCM melting, are very weak in the bottom part of the cavity compared to its upper part. It can be seen that PCM melting occurs faster in the case ω*_na_* = 0.06, which is due to conduction heat transfer enhancement at the early stage of melting. It should be noted that the presence of nanoparticles increases the dynamic viscosity of liquid PCM and reduces the strength of natural convection flows at the final stages of melting. This observation is further confirmed in Figure 7, which illustrates the evaluation of the melted volume fraction MVF and the stored energy ES as functions of time for different values of ω*_na_*. In the charging process, both the MVF and the ES increase for higher values of ω*_na_*. Moreover, full melting (MVF = 1) is achieved faster when ω*_na_* is increased. This is due, as previously explained, to the enhanced thermal conductivity when nanoparticles are added to the PCM, and the growth of dynamic viscosity. The stored energy at 750 min is slightly larger for pure PCM compared to NePCM, which is mainly due to the higher overall latent heat of pure PCM. The presence of nanoparticles reduces PCM’s overall latent heat since they cannot store latent heat. They also slightly increase the heat capacity of the PCM. However, the increase of heat capacity contributing to the sensible heat is much lower than the change in the latent heat. Thus, by using nanoparticles, the ultimate value of stored energy can be reduced slightly.

Figure 7c displays the thermal resistance (*R_NP_*) at the NePCM side for various nanoparticle concentrations. Generally, *R_NP_* increases as the melting advances. By the advancement of melting, the distance of the cold surface (melting front) increases from the heated surface (wavy surface). The increase of distance increases the thermal resistance. This figure shows that at the beginning of melting, where the heat transfer is conduction dominant, the increase of volume fraction of nanoparticles decreases the thermal resistance, *R_NP_*. About 300 min, there is a turning point that the thermal resistance grows as the volume fraction of nanoparticles rises. Figure 7a shows about 80% melting at 300 min. Thus, at 300 min, a significant amount of liquid PCM exists in the enclosure, and the natural convection heat transfer is significant (see Figure 6). Therefore, nanoparticle presence increases the dynamic viscosity and reduces the strength of natural convection leading to the growth of thermal resistance.

**Figure 8:**
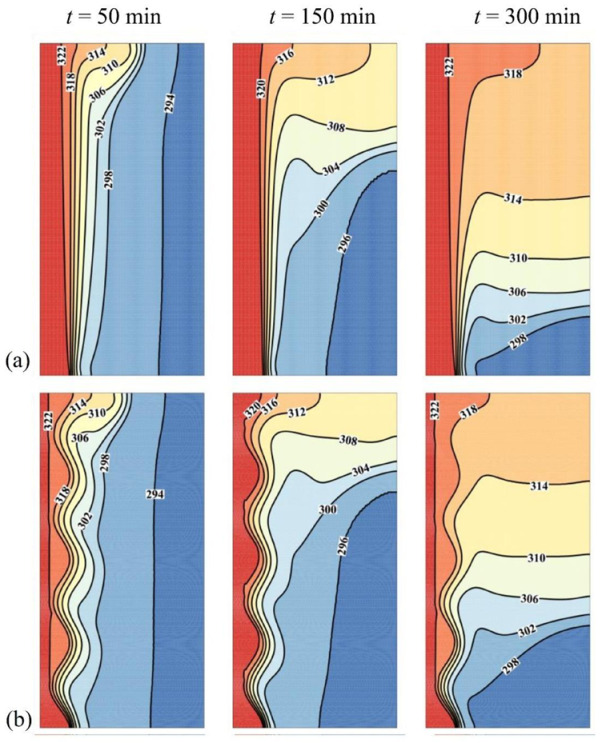
Comparison between contours of isotherm in different wave amplitude (*A*), (**a**): optimum case with *A* = 0 mm; (**b**): Case 6 with *A* = 3 mm, for ω*_na_* = 0.06, and *N* = 4.

**Figure 9:**
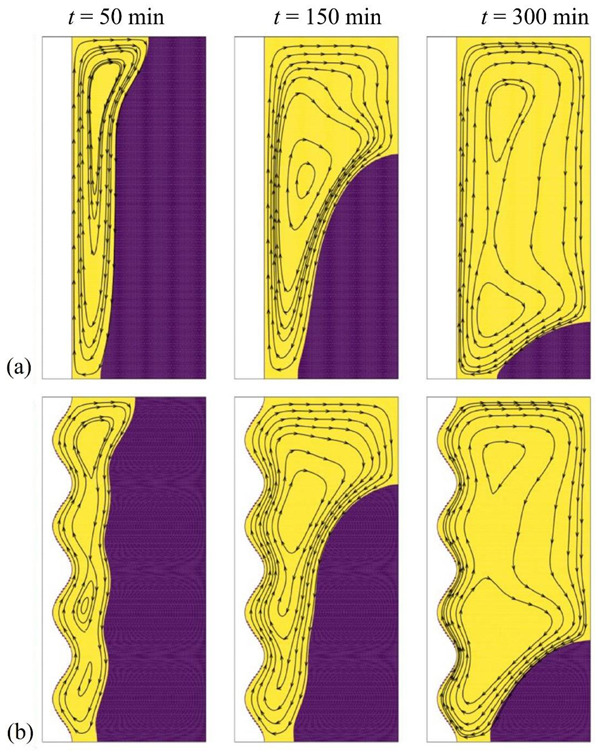
Effect of wave amplitude (*A*) on the streamlines, (**a**): optimum case with *A* = 0 mm; (**b**): Case 6 with *A* = 3 mm, for ω*_na_* = 0.06, and *N* = 4.

The effect of the hot tube amplitude on the temperature distribution and on the flow-patterns is illustrated in Figure 8 and Figure 9. Two amplitudes are considered: *A* = 0 (flat tube), which corresponds to the optimal case, and *A* = 0.06. It is clear that the streamlines mimic the wavy tube profile in the left portion, where heat is mainly transmitted by conduction. This behavior remains when time passes. However, it is more apparent in the bottom part of the cavity, as convective effects dominate the PCM behavior in the upper part. For Cases 4–6, the steady-state pressure drop was computed as 2.01 × 10^−4^ Pa (*A* = 1 mm), 6.63 × 10^−3^ Pa (*A* = 2 mm), and 6.53 10^−2^ Pa (*A* = 3 mm). The pressure drop of the optimum case is 3.55 × 10^−5^ Pa (*A* = 0 mm). As seen, there is an exponential pressure drop by the increase of undulation amplitude.

For a fixed *N* and *A*, the values of *R_HTF_* were changed minimally during the phase change. This is since the HTF fluid-flow is independent of the melting process. The slight changes are from the local variation of the average temperature of the tube during the phase change. The average values of *R_HTF_* were found as 0.63 K/W (*A* = 1 mm), 0.73 K/W (*A* = 2 mm), 0.65 K/W (*A* = 3 mm). The *R_HTF_* resistance for the optimum design (straight tube) was 0.5 K/W, which is smaller than all wavy cases.

**Figure 10:**
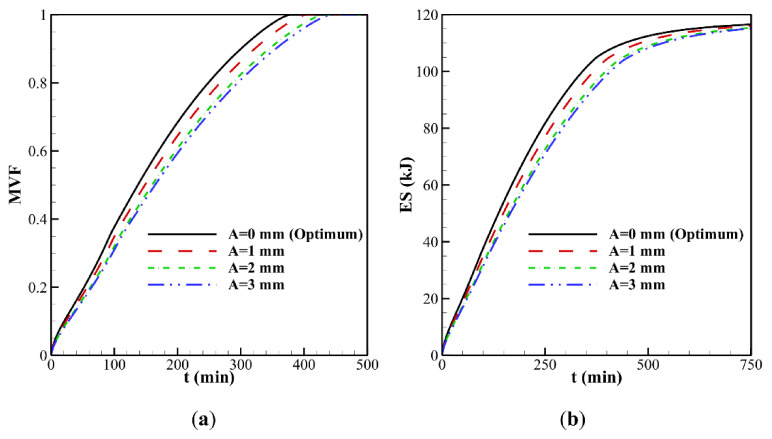
Effect of wave amplitude (*A*) for ω*_na_* = 0.06 and *N* = 4: (**a**) melting volume fraction; (**b**) total energy storage.

The waviness of the tube increases the distance between the heated line and the PCM and slows down its melting compared to a flat tube. Moreover, the waves act as obstacles that trap the convective flows in the molten region of the enclosure. Thus, the increase of surface contact is not as effective as the suppression of natural convection flow. This appears in the size of the thermal layer near the tube, which is greater for *A* = 0. In the upper part of the cavity, this behavior is less effective as the convective effects are dominant. On the other hand, PCM melts faster in the bottom part in the case of a flat-tube. This results in an overall faster PCM melting and a greater volume of liquid PCM for *A* = 0. For these reasons, it is clear that reducing *A* shows a positive effect on both MVF and ES as indicated in Figure 10.

**Figure 11:**
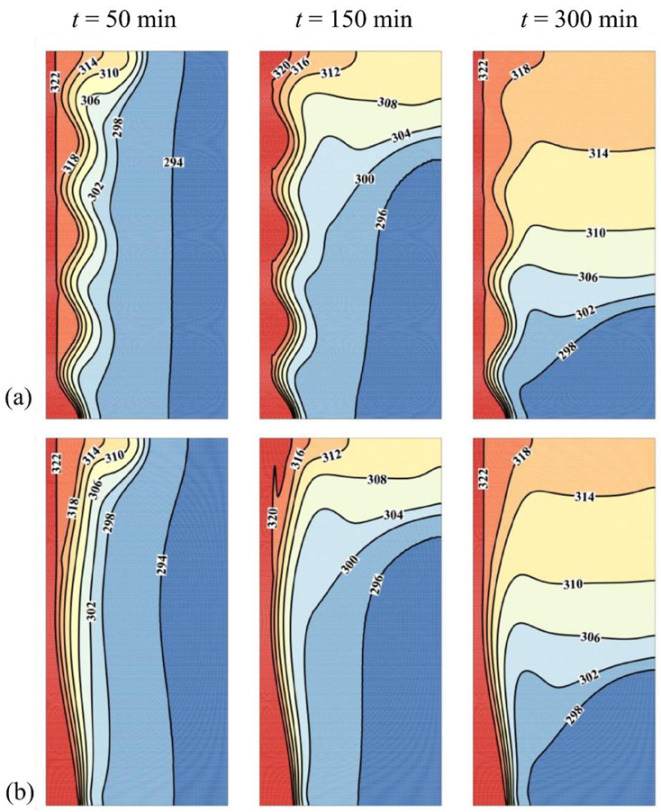
Effect of number of waves (*N*) on the contours of isotherms, (**a**) Case 6 with *N* = 4 and ω*_na_* = 0.06; (**b**) Case 7 with *N* = 1 and ω*_na_* = 0.06.

**Figure 12:**
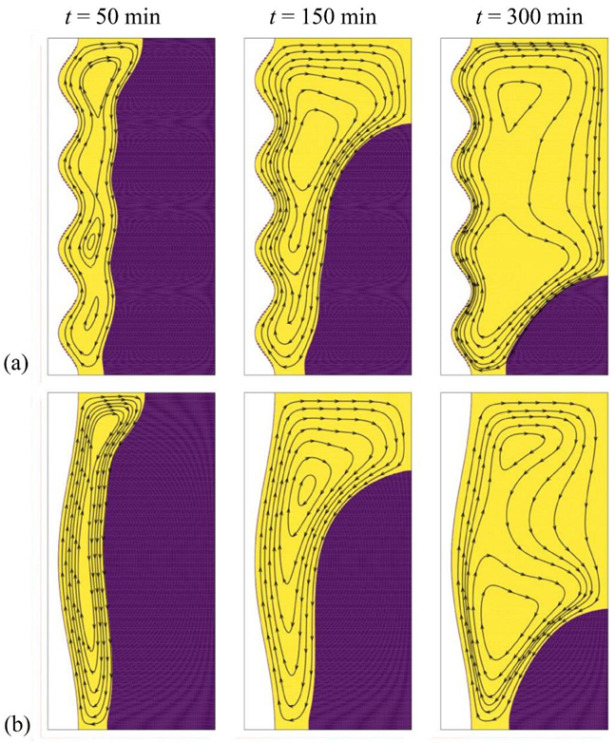
Effect of number of waves (*N*) on the streamlines, (**a**) Case 6 with *N* = 4 and ω*_na_* = 0.06; (**b**) Case 7 with *N* = 1 and ω*_na_* = 0.06.

**Figure 13:**
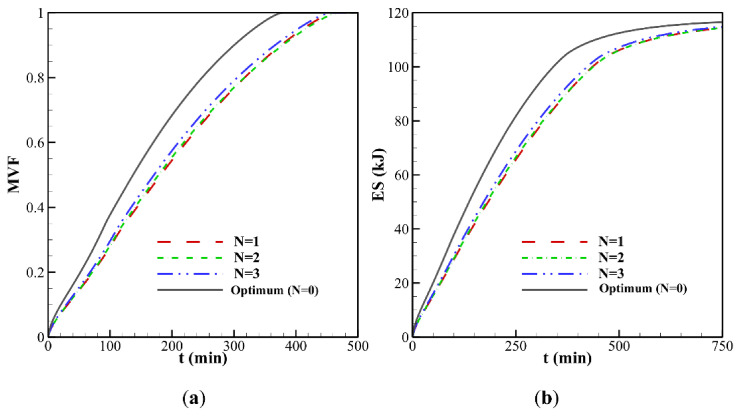
Effect of number of waves (*N*) for ω*_na_* = 0.06 and *A* = 3 mm: (**a**) melting volume fraction; (**b**) total energy storage.

The last control factor to be assessed is the number of undulations *N* of the wavy tube. The thermal and flow patterns of the PCM in the enclosure are shown in Figure 11 and Figure 12 for two values of *N*, *N* = 4 (the optimal value) and *N* = 1, and for the highest wave amplitude of 3 mm. The flow streamlines mimic the wall profile near the hot tube, as the number of oscillations of the streamlines is equal to that of the tube. The volume of melted PCM, as well as the temperature in different zones of the cavity, are larger for *N* = 4 compared to *N* = 1, which can be attributed to the increase of surface contact between the PCM and the hot tube when the same amplitude is considered. This results in an increase of the MVF and the ES when *N* is raised, as observed in Figure 13. However, the presence of undulation entraps the fluid flow at the tube side and reduces the heat transfer. Thus, on the one hand, the presence of *N* contributes to the increase of surface heat transfer, and on the other hand, it reduces the temperature gradients and the intensity of heat transfer.

The analysis of the flow patterns and the isotherms in the enclosure, as well as of the MVF and ES time evolution, confirm the optimization of the design for the determined values of the control factors. For Cases 7–9, the steady state pressure drop was computes as 0.082 Pa (*N* = 1), 0.080 Pa (*N* = 2), and 0.072 Pa (*N* = 3), 3.55 × 10^−5^ Pa (the optimum case *N* = 0). As seen, there is a sharp pressure drop in the presence of undulation compared to the case of a straight tube. The pressure drop reduces as the number of undulations increases. The average values of *R_HTF_* were found as 0.64 K/W (*N* = 1), 0.72 K/W (*N* = 2), 0.70 K/W (*N* = 3).

## 5. Conclusions

In the present numerical study, an LHTES unit’s design based on a wavy tube and NePCM was optimized, based on Taguchi’s approach. The control factors considered were the amplitude *A* of the wavy tube and its number of undulations *N*, and the volume fraction of the nanoparticles ω*_na_*.

- Each control factor was assigned four different values corresponding to four levels. The L16 orthogonal array method was used to reduce the number of required tests. The time required to melt the PCM fully was selected as the optimization result, so the design was optimized based on: the lower the full melting time, the better the result.
- It was found that the optimal values of the control factors are: ω*_na_* = 0.06 and a plane tube with no undulation (*A* = 0 and *N* = not applicable). Conducting further simulations with values around the optimal ones confirmed that the melting time is the lowest when the optimal values are selected. Plotting the isotherms and the streamlines for various values of the control factors showed that reducing the amplitude improves heat transfer in the bottom region and enhances PCM melting and energy storage.
- Increasing the nanoparticle fraction elevates the thermal conductivity of the PCM and enhances heat transfer, resulting in an accelerated melting and high stored energy. Finally, increasing the number of undulations of the wavy tube increases the heated contact surface with the PCM, which leads to a faster PCM melting and improves stored energy.

## Figures and Tables

**Table 1:** Properties of LHTES materials [24,25].

Properties	Coconut Oil (Measured)		Heat Transfer Fluid (HTF) (Water at *T_in_*)	CuO
	Liquid (32 °C)	Solid (15 °C)		
*k* (W m^−1^ K^−1^)	0.166 ± 1.2%	0.228	0.623	18
*T_me_* (°C)	-	24 (± 1 °C)	-	-
μ (Pa s)	0.0326 ± 3%	-	0.705 × 10^−3^	-
ρ (kg × m^−3^)	914 ± 0.11%	920	993.73	6500
*h_f_* (kJ × kg^−1^)	103 ± 1%	-	-	-
*C_p_* (J kg^−1^ × K^−1^)	2010 ± 0.2%	3750	4178	540

**Table 2:** Grid independency assessment in dimensional time of 20,000 s.

Grid Size		Melted Volume Fraction in 20,000 s	Error Percentage (%)
Fluid Domain (in pipe)	PCM Domain		
15 × 75	75 × 75	0.80819	-
20 × 100	100 × 100	0.80246	0.71
25 × 125	125 × 125	0.79932	0.40
30 × 150	150 × 150	0.79808	0.16
40 × 200	200 × 200	0.79682	0.16

* The marked case was chosen for numerical computations of the results.

**Table 3:** The range and levels of control parameters.

Factors	Description	Level 1	Level 2	Level 3	Level 4
**A**	Nanoparticle concentration, ω*_na_*	0	0.02	0.04	0.06
**B**	Wave amplitude *A* (mm)	0	1	2	3
**C**	Number of waves, N	1	2	3	4

**Table 4:** Taguchi 16 orthogonal table corresponding to range and levels of control parameters.

Test Number	Control Parameters			Full Melt Time	Signal to Noise (S/N) Ratio
	ω_na_	*A* (mm)	*N*		
**1**	0.00	0	1	26,500	−88.4649
**2**	0.00	1	2	28,287	−89.0317
**3**	0.00	2	3	30,512	−89.6894
**4**	0.00	3	4	31,112	−89.8586
**5**	0.02	0	2	25,149	−88.0104
**6**	0.02	1	1	26,636	−88.5094
**7**	0.02	2	4	28,639	−89.1392
**8**	0.02	3	3	30,363	−89.6469
**9**	0.04	0	3	23,893	−87.5654
**10**	0.04	1	4	25,476	−88.1226
**11**	0.04	2	1	27,178	−88.6843
**12**	0.04	3	2	29,587	−89.4220
**13**	0.06	0	4	22,709	−87.1240
**14**	0.06	1	3	24,197	−87.6752
**15**	0.06	2	2	26,320	−88.4057
**16**	0.06	3	1	27,750	−88.8606

**Table 5:** The optimum values of the controlling parameters.

Optimum Factors			Optimum Time of Melting
ω*_na_*	*A* (mm)	*N*	
0.06	0	4	22,709 s

**Table 6:** The rank values of the controlling parameters for S/N.

Levels	ω*_na_*	*A* (mm)	*N*
**1**	−89.26	−87.79	−88.63
**2**	−88.83	−88.33	−88.72
**3**	−88.45	−88.98	−88.64
**4**	−88.02	−89.45	−88.56
**Δ**	1.24	1.66	0.16
**Rank**	2	1	3

**Table 7:** Further investigation on the impact of design parameters around the optimum point for CNTs.

Case	Parameter Investigation	A	B	C	Melting Time		
		ω*_na_*	*A* (mm)	N	80% Melt	90% Melt	Full Melt
1	ω*_na_*	0.0	0	4	17,550	21,150	26,550
2		0.02	0	4	16,650	20,100	25,200
3		0.04	0	4	15,750	19,050	24,000
4	*A*	0.06	1	4	16,050	19,350	24,300
5		0.06	2	4	17,250	20,700	25,950
6		0.06	3	4	17,700	21,450	26,700
7	*N*	0.06	3	1	19,050	22,650	27,750
8		0.06	3	2	19,050	22,800	28,200
9		0.06	3	3	18,450	22,050	27,450

## Data Availability

Data is contained within the article.

## Sample Availability

Samples of the compounds are not available from the authors.
